# Development of Sustainable Engineered Cementitious Composites by Incorporating Local Recycled Fine Aggregate

**DOI:** 10.3390/polym15122701

**Published:** 2023-06-16

**Authors:** Yingwu Zhou, Wenhui Guo, Shuyue Zheng, Feng Xing, Menghuan Guo, Zhongfeng Zhu

**Affiliations:** 1 Guangdong Provincial Key Laboratory of Durability for Marine Civil Engineering, Shenzhen University, Shenzhen 518060, China; ywzhou@szu.edu.cn (Y.Z.); 2060471040@email.szu.edu.cn (W.G.); 1810332085@email.szu.edu.cn (S.Z.); xingf@szu.edu.cn (F.X.); zhongfeng.zhu@szu.edu.cn (Z.Z.); 2 Beijing Urban Construction Design and Development Group Co., Ltd., Beijing 100037, China

**Keywords:** engineered cementitious composites, limestone calcined clay cement, recycled fine aggregate, mechanical properties, environmental impact

## Abstract

In this study, sustainable engineered cementitious composites (ECC) exhibiting high tensile strength as well as high tensile strain capacity were successfully developed by incorporating polyethylene (PE) fiber, local recycled fine aggregate (RFA), and limestone calcined clay cement (LC^3^). The improvement in tensile strength and tensile ductility was attributed to the self-cementing properties of RFA as well as the pozzolanic reaction between calcined clay and cement. Carbonate aluminates were also generated owing to the reaction between calcium carbonate in limestone and the aluminates in both calcined clay and cement. The bond strength between fiber and matrix was also enhanced. At the age of 150 days, the tensile stress-strain curves of ECC containing LC^3^ and RFA shifted from a bilinear model to a trilinear model, and the hydrophobic PE fiber exhibited hydrophilic bonding performance when embedded in RFA-LC^3^-ECC matrix, which could be explained by the densified cementitious matrix as well as the refined pore structure of ECC. Moreover, the substitution of ordinary Portland cement (OPC) by LC^3^ resulted in energy consumption and equivalent CO_2_ emission reduction ratios of 13.61% and 30.34%, respectively, when the replacement ratio of LC^3^ is 35%. Therefore, PE fiber-reinforced RFA-LC^3^-ECC demonstrates excellent mechanical performance as well as considerable environmental benefits.

## 1. Introduction

Engineered cementitious composites (ECC) are a type of fiber-reinforced cementitious composite material with high ductility and toughness [1,2]. They are mainly composed of cement, mineral admixtures, fine aggregates, water, superplasticizers, and up to 2% fibers [3,4]. The use of materials such as silica fume and fly ash enhances the impermeability and durability of ECC [5], while the low water-cement ratio improves the bond between cement, fine particle materials, and chemical additives [6]. Based on micromechanical principles, researchers have designed ECC mixtures that undergo strain hardening after cracking and exhibit highly improved ductility compared to normal concrete [7]. The tensile strain capacity of ECC can reach up to 8% under different environmental conditions [8,9,10,11] and with different types of fibers and additives [12,13,14]. Moreover, studies have shown that ECC demonstrates good durability and self-healing ability in comparison to concrete [15], owing to the generation of significantly tight microcracks.

The first utilization of PE fibers in the production of ECC was pioneered by Li et al. [16]. PE fibers play a crucial role in fiber-reinforced cementitious composites by offering high strength, lightweight, and corrosion resistance. They effectively enhance the tensile strength and toughness of cementitious materials, preventing crack propagation and damage, thus, improving durability and impact resistance. Additionally, incorporation of PE fibers reduces the shrinkage rate of cementitious materials, mitigating the risk of cracking. PE fibers find wide applications in various fields, including construction, roads, and water infrastructure, providing vital support to enhance the performance of fiber-reinforced cementitious materials. In addition to PE fibers, researchers will also utilize polyvinyl alcohol fibers (PVA) to produce (ECC). The main difference between these two types of fibers is that PVA fibers are hydrophilic, while PE fibers are hydrophobic. The strong bond between PVA fibers and cementitious materials can lead to premature fiber rupture, affecting the ductility of PVA-ECC. To mitigate the bonding strength between the fibers and the matrix, it is common practice to apply oil coating on the surface of PVA fibers. PE fibers, however, are inherently hydrophobic and do not require oil treatment on their surface.

Despite its relatively superior performance, traditional ECC uses ordinary cement as binding material and quartz sand as fine aggregate. However, the production process of cement is energy intensive, and the depletion crisis of natural construction resources prompts the need to find suitable environmentally friendly alternatives for cement and quartz sand. To alleviate the environmental pressure caused by ECC production, the use of limestone calcined clay cement (LC^3^) as a replacement for ordinary Portland cement (OPC) and the use of recycled fine aggregate (RFA) as a replacement for quartz sand could be a prospective solution.

LC^3^ is a new type of cement proposed by Scrivener et al. [17] that utilizes calcined clay with pozzolanic activity and limestone as effective fillers to replace OPC. By using LC^3^, CO_2_ emissions are reduced up to 30%, and the energy consumption is reduced by 15% to 20% compared to traditional cement [17]. Industrial trials conducted in Cuba and India have demonstrated that LC^3^ made by mixing only 50% cement clinker, calcined clay, and limestone has mechanical properties equivalent to CEM I Portland cement with a clinker content of over 90% [18]. Currently, some researchers have explored the possibility of incorporating LC^3^ into strain-hardening cementitious composites. Zhu et al. [19] successfully developed LC^3^-ECC with a tensile strain of up to 6% using LC^3^ and PP fibers. Compared with the control group, the material cost decreased by 61%, the energy consumption decreased by 45%, and carbon emissions decreased by 48%. Zhang et al. [20] prepared LC^3^-ECC with a tensile strain of up to 6% and an average crack width of less than 50 μm. The volume fraction of pores larger than >100 nm was also reduced by using LC^3^. Compared to traditional ECC and concrete, the CO_2_ emissions of LC^3^-ECC were reduced by 32% and 28%, respectively.

Moreover, RFA is a byproduct of crushed waste concrete, composed of original aggregate and a large proportion of adhered old mortar [21,22]. It has an irregular shape [23] and a relatively porous microstructure [24,25]. Poon et al. [26] studied the effect of RFA on unbound subbase, while Silva et al. [27] investigated its effect on fresh concrete. The studies have shown that due to the presence of unhydrated cement in the old mortar adhered to original aggregate, particles with a diameter smaller than 0.15 mm and between 0.3–0.6 mm exhibit obvious self-cementing properties. Li and Yang first attempted to incorporate RFA into ECC [28]. The results indicated that appropriate mix design of the composite material could achieve good compressive strength and strain capacity. As the morphology of sand directly affects the strength and strain capacity of ECC, strength improvement is achieved by reducing the particle roundness and the sphericity of agglomerates [29]. In fact, other types of waste materials, including glass powder [30,31,32] and coal bottom ash [33], can also be utilized as recycled aggregates, among others [34,35]. These materials contribute to sustainable practices by diverting waste from landfills and reducing the environmental impact of construction activities. Despite ongoing research on the use of RFA in ECC [36,37], the mechanical performance of ECC made with RFA still needs to be improved. In recent years, the development of the construction industry has raised the demand for high performance concrete. Thus, further research concerning the high-quality usage of RFA, such as the development of high-strength and high-toughness ECC by incorporating RFA, is highly needed.

This work aims to develop sustainable, high-performance ECC by using environmentally friendly LC^3^ and locally available RFA. The carbon and energy footprints of ECC are remarkably reduced, and the environmental pressure of waste disposal is alleviated. In addition to its obvious environmental benefits, the developed ECC features excellent mechanical performance. Firstly, the compressive strength and uniaxial tensile behavior of RFA-LC^3^-ECC were investigated. The influence of curing age on the mechanical performance of ECC was also studied. Then, the interfacial bonding behavior between fibers and ECC matrix was investigated. The hydration products of ECC matrix were characterized by carrying out X-ray diffraction (XRD) analysis. Finally, the environmental impact analysis of RFA-LC^3^-ECC was evaluated, and the comprehensive performance of the developed ECC was discussed.

## 2. Materials and Methods

### 2.1. Materials and Mixture Proportion

**Figure 1:**
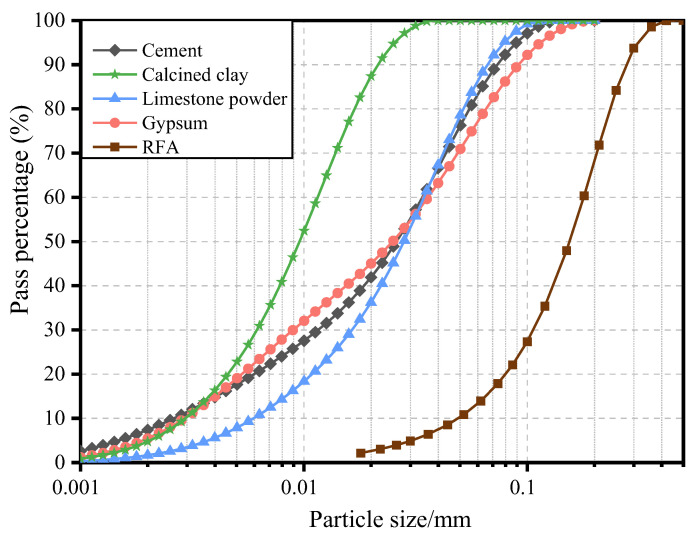
Grading curves of the ingredients.

**Figure 2:**
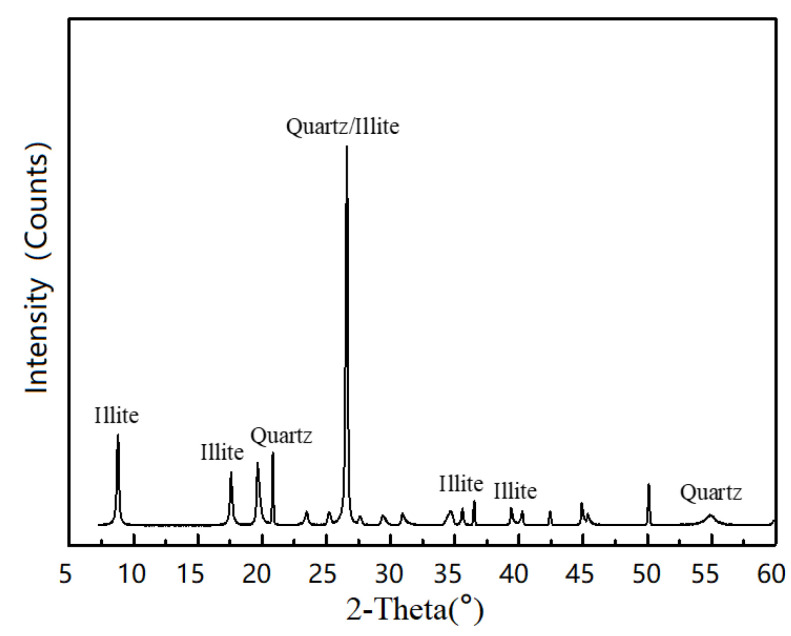
X-ray diffraction pattern of calcined clay.

**Figure 3:**
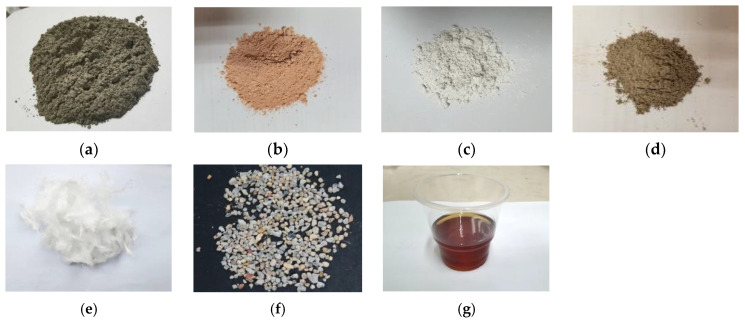
Materials utilized in this study: (**a**) cement; (**b**) calcined clay; (**c**) limestone; (**d**) gypsum; (**e**) PE fiber; (**f**) RFA; (**g**) HRWRA.

The control mix of ECC used in this study included OPC of grade 52.5R, RFA, Polyethylene (PE) fibers, Sika Viscocrete 3301-40 High Range Water Reducing Agent (HRWRA), and water. For the LC^3^-ECC test groups, calcined clay and limestone powder were used to replace OPC at a ratio of 2:1 for the ECC-LC^3^ group, with replacement ratios of 35% and 50%, respectively, as reported in [38]. The design of mix proportion is shown in Table 1. Gypsum was used to regulate the early reaction of aluminum salts. The effective water-to-binder ratio for all mixtures in this study was 0.24. The particle size distribution of the raw materials was determined using a laser particle size analyzer, and the results are shown in Figure 1. The chemical compositions of the raw materials used in the study were quantitatively analyzed using X-ray fluorescence (XRF) analysis with a ZSX Primus II X-ray fluorescence spectrometer (Rigaku, Tokyo, Japan), and the results are summarized in Table 2. The calcined clay used in this study was obtained from the kaolin tailings in Maoming, China, and mainly consisted of quartz, illite, and amorphous phases, as evidenced by the XRD pattern shown in Figure 2. The apparent densities of cement, calcined clay, and limestone were 3140 kg/m^3^, 1820 kg/m^3^, and 2710 kg/m^3^, respectively. The morphology of the used materials is shown in Figure 3. The RFA used in this study was obtained from a recycling plant in Shenzhen, China. The apparent density of RFA is 2408 kg/m^3^, and the water absorption ratio is 10.2%. The Polyethylene (PE) fibers were purchased from QUANTUMETA in Beijing, China, and their detailed properties are listed in Table 3.

Below are the details of the mixing process adopted to ensure the uniform distribution of fibers in the ECC mixture. First, the solid mixture, including OPC, calcined clay, limestone powder, gypsum, and quartz sand, was mixed at low speed for 3 min in a mixer. Then, water and HRWRA were added to the dry mixture, and the mixture was stirred at low speed for 5 min, followed by high-speed stirring for 1 min while gradually adding PE fibers. Finally, the mixture was stirred for one additional minute to eliminate fiber agglomeration. The slump of the fresh ECC mixture was tested according to the standard GB/T 2419-2005. The specimens were demolded after 24 h and transferred to a curing room with a temperature of 23 ± 3 °C and a relative humidity of 95% for a curing period of 28 days.

### 2.2. Macroscopic Mechanical Testing

**Figure 4:**
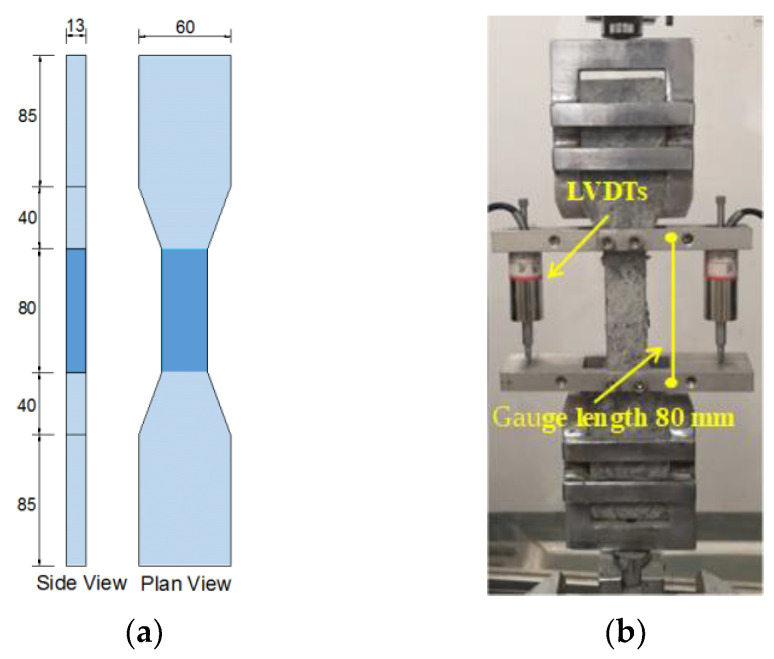
Dog-bone shaped specimen for tensile test: (**a**) geometric size; (**b**) test instrument. (unit: mm).

In this study, the compressive strength test of the ECC test group was conducted according to the JGJ/T20-2009 standard. Three 50 × 50 × 50 mm^3^ cubic specimens were used for each group, and the MTS 200T type compression testing machine was used. The loading rate was set to 0.3 MPa/s. According to the recommendation of the Japan Society of Civil Engineers (JSCE) [39], the dog bone-shaped specimen was used to test the tensile properties of ECC, and the geometric dimensions of the specimen are shown in Figure 4a. The MTS Landmark electro-hydraulic servo machine was used for uniaxial tensile testing with a loading rate of 1 mm/min. Two linear variable displacement transducers (LVDTs) were installed on both sides of the specimen to measure the tensile deformation of the specimen within a length range of 80 mm, as shown in Figure 4b.

### 2.3. Single Fiber Pullout Test

**Figure 5:**
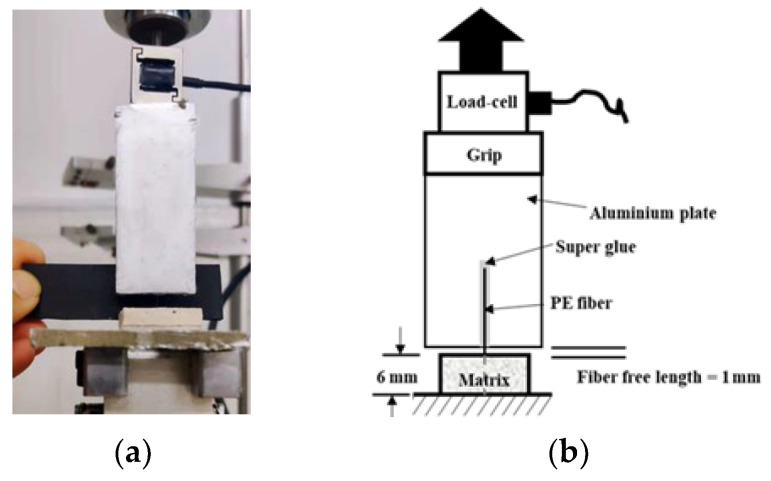
Setup for single fiber pullout test: (**a**) actual photo; (**b**) schematic diagram.

In this study, a single fiber pullout test was used to characterize the interfacial properties between PE fibers and LC^3^-based cementitious matrix. Following the specimen preparation method of Lu et al. [40], a single PE fiber was glued onto a plastic mold and ECC mortar matrix was cast. After 24 h, the specimen was taken out and cured for 28 days at room temperature. By cutting perpendicular to the fiber length direction, a single fiber pullout sample was obtained. The fiber embedment length in the matrix was set at 6 mm and the fiber-free length was 1 mm. The test was conducted using a REGER tensile testing machine, with a load cell of 5 N and a loading rate of 0.5 mm/min. To fix the polyethylene fiber, a strong adhesive was used to attach the fiber to a smooth aluminum plate, and the displacement was provided by the machine. The test setup is shown in Figure 5a and a schematic diagram of the test details is shown in Figure 5b.

### 2.4. X-ray Diffraction Analysis

XRD tests were conducted to analyze the mineral composition of the hydration products in the LC^3^-ECC matrix of the recycled aggregate in this study. Prior to testing, the ECC matrix sample particles were immersed in anhydrous ethanol to stop the hydration reaction. After vacuum drying at 60 °C for 24 h, the samples were ground into fine powder. The XRD tests were carried out using a D8 ADVANCE X-ray diffractometer from Bruker (Karlsruhe,, Germany). Cu-Kα (λ = 1.54 Å) was used as the test radiation, with a voltage of 40 kV, a current of 40 mA, a step size of 0.01, and a scan time of 0.3 s.

### 2.5. Methodologies of Environmental Impact Assessment

The environmental impact of the production process of ECC incorporating LC^3^ and recycled fine aggregate was evaluated using the LCA method in this study. The LCA evaluation method referred to the ISO 14040 standard, and the commercial LCA analysis software GaBi (10.5.1.124, Sphera Solutions GmbH, Leinfelden-Echterdingen, Germany) was used as the analysis tool. The calculation data for carbon emissions and energy consumption in this study were obtained from the GaBi software database and the relevant reference literature (Table 4). After building the model in the software, the CO_2_ emissions (GWP) and energy consumption (PED) of the materials can be calculated.

## 3. Results and Discussions

### 3.1. Flowability

The results of flowability tests for the fresh mixes of the three types of recycled aggregate ECC used in this study are presented in Table 5. As the amount of added LC^3^ increased, the average value of flowability gradually decreased in the ECC mixtures containing LC^3^. This is due to the fact that the particle size of calcined clay is smaller than that of cement, leading to an increased surface area and greater adsorption of water molecules in the LC^3^ system, and resulting in a higher water demand and reduced flowability. Compared to the natural aggregate LC^3^-ECC system studied by Gong et al. [43], the slump flow of the recycled aggregate LC^3^-ECC was further reduced. This can be attributed to the finer particle size and higher specific surface area of the RFA, as well as its higher water absorption, which led to a further decrease in the flowability.

### 3.2. Compressive Strength

**Figure 6:**
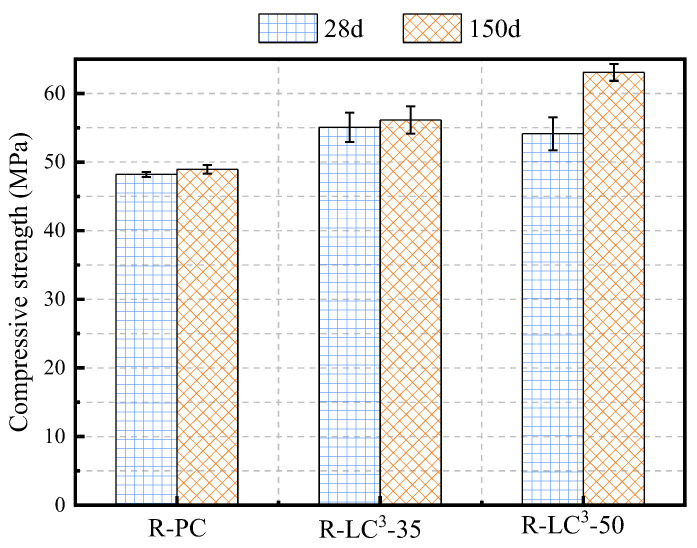
Compressive Strength.

In this section, the average compressive strengths of three types of recycled aggregate ECC at different ages were studied. As shown in Figure 6, the compressive strengths of R-LC^3^-35 and R-LC^3^-50 at 28 days were 55.07 MPa and 54.13 MPa, respectively, which were 14.2% and 12.3% greater than that of the PC group. At 150 days, the compressive strengths of R-LC^3^-35 and R-LC^3^-50 were 56.13 MPa and 63.08 MPa, respectively, which were 14.7% and 28.9% greater than that of the PC group. The reason for the increase in compressive strength of LC^3^-replaced cementitious group may be that the calcined clay undergoes pozzolanic reaction and synergistically generates hydration gels, which densifies the cementitious matrix, and thus, improves the compressive strength. In addition to the physical filling effect [44], the synergistic effect of limestone and calcined clay leads to the generation of carbonate aluminates, which also benefits for the improvement of compressive strength.

### 3.3. Tensile Properties

**Figure 7:**
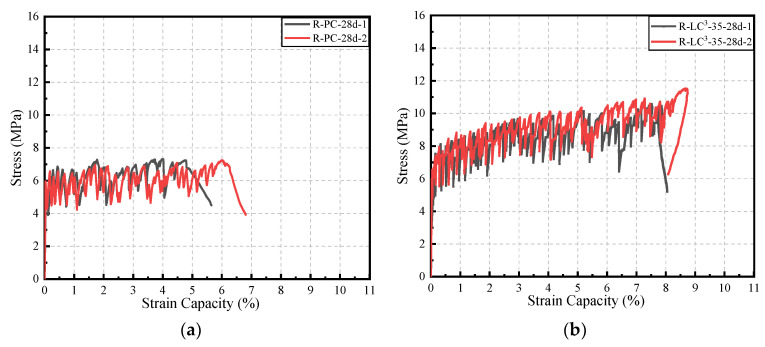

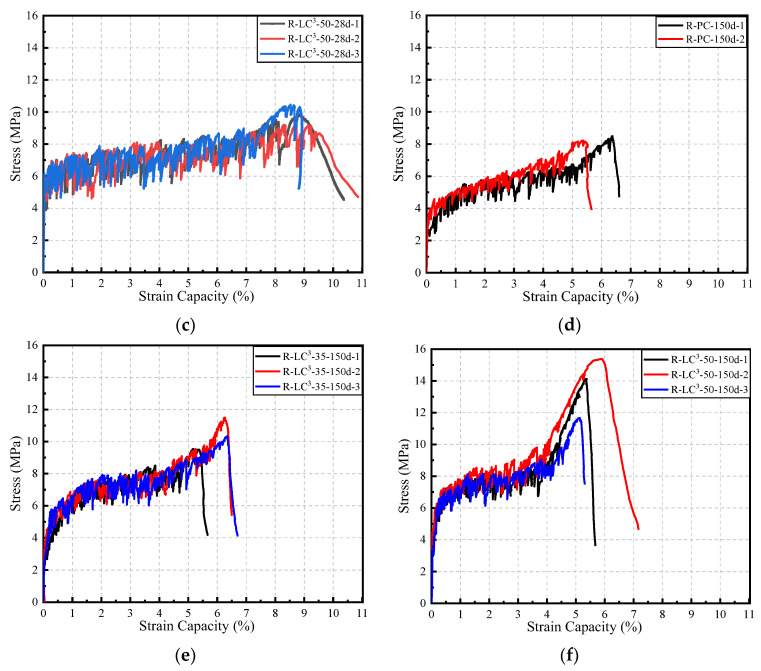
Tensile stress-strain curves: (**a**) R-PC-28d; (**b**) R-LC^3^-35-28d; (**c**) R-LC^3^-50-28d; (**d**) R-PC-150d; (**e**) R-LC^3^-35-150d; (**f**) R-LC^3^-50-150d.

The tensile stress-strain curves of the three kinds of ECC are presented in Figure 7. At the age of 28 days, the stress-strain relationship of recycled aggregate ECC specimens could be described by a bilinear model. Three stages included the initial developing stage, the strain hardening stage, and the failure stage. When the age reached 150 days, the stress-strain curve of LC^3^-ECC incorporating recycled aggregate was closer to a trilinear model, with a second strain hardening stage appearing. At this stage, the cement matrix had completely lost its bearing capacity, and no new cracks appeared. All stress was borne by the fibers, and the width of the original cracks continued to increase until the stress value reached its limit. During the loading process, the stress fluctuated slightly due to the pulling out or breaking of fibers. Unlike the first strain hardening stage, the stress-strain curve in this stage was close to a straight line, and the slope was significantly larger than that of the first strain hardening stage. The reason for the trilinear strain hardening phenomenon may be that PE fibers are hydrophobic materials, but after 28 days, the hydration reaction in the LC^3^ matrix continues, making the bond between LC^3^ and PE fibers tighter than that between ordinary cement and PE fibers. This results in the formation of a trilinear hardening model. More fibers are broken, instead of being pulled out, which is one of the reasons for the decrease in ductility at the age of 150 days. In addition, previous studies have shown that the addition of LC^3^ can lead to refinement of the pore size and reduction of porosity in the cement matrix, which also helps to enhance the bonding performance between fibers and matrix [43].

**Figure 8:**
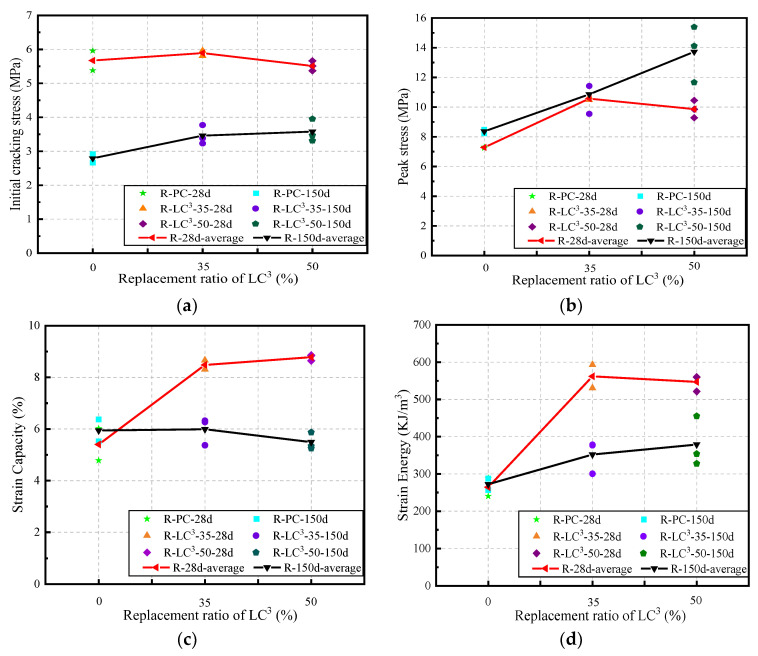
Tensile parameters: (**a**) Initial cracking stress; (**b**) Peak stress; (**c**) Strain capacity; (**d**) Strain energy.

Based on the tensile stress-strain curves, the tensile parameters of R-LC^3^-ECC at different ages and substitution ratios were analyzed, as shown in Figure 8. At the age of 28 days, the average initial cracking stress was similar for the three formulations, with R-PC at 5.67 MPa, R-LC^3^-35 at 5.89 MPa, and R-LC^3^-50 at 5.51 MPa (Figure 8a). The average peak stress of R-PC, R-LC^3^-35, and R-LC^3^-50 was 7.28 MPa, 10.40 MPa, and 9.86 MPa, respectively (Figure 8b). The introduction of LC^3^ contributes to the improvement of tensile strength. In terms of the tensile strain capacity, it increased from 5.5% for R-PC to 8.48% for R-LC^3^-35 and 8.78% for R-LC^3^-50 (Figure 8c). This is due to the addition of LC^3^, which contributes to the generation of more C-S-H and C-A-S-H gels in the ECC system and enhances the bond strength between fiber and matrix. The self-cementing properties of RFA also favorably lead to the formation of additional hydration products. Furthermore, the addition of LC^3^ significantly increased the strain energy (Figure 8d). Compared to the reference group with strain energy of 264 kJ/m^3^, the strain energy of R-LC^3^-35 and R-LC^3^-50 was 113% and 107% greater, respectively. It could be concluded that the tensile strength and tensile ductility of the R-LC^3^ groups were improved, indicating that the combination of LC^3^ and recycled aggregate demonstrates a positive effect on the properties of LC^3^-ECC.

**Figure 9:**
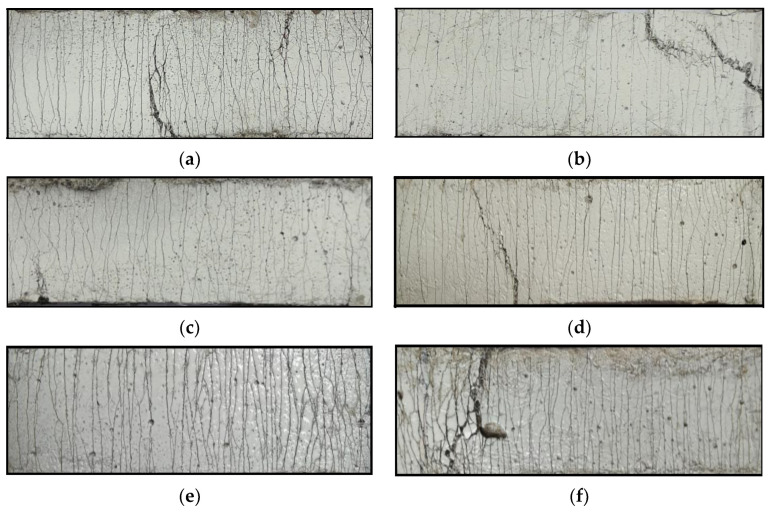
Crack patterns of the three types of ECC after the rupture of the specimen: (**a**) R-PC-28d; (**b**) R-LC^3^-35-28d; (**c**) R-LC^3^-50-28d; (**d**) R-PC-150d; (**e**) R-LC^3^-35-150d; (**f**) R-LC^3^-50-150d.

The crack patterns of the dog bone specimens after failure are shown in Figure 9. All specimens exhibited multiple cracking characteristics. Dense and homogeneous cracks were formed during the tensile loading process. At ages of 28 and 150 days, the R-LC^3^-50 had the highest number of cracks, while R-PC had the lowest. In addition, the number of cracks in the 150-day-old dog bone specimens was generally higher than that in the 28-day-old specimens. The average number of cracks, *N_c_*, average crack width, *W_c_*, and average crack spacing, *S_c_*, on both sides of the center of the dog bone with a gauge length of 80 mm were calculated for each specimen, and the results are summarized in Table 6. The value of *W_c_* was determined by dividing the elongation of the central area of the dog bone specimen by the number of cracks, while the value of *S_c_* was determined by dividing 80 mm by the number of cracks. According to the data in Table 6, the addition of LC^3^ increased the number of cracks, and a decrease in the average crack width and spacing was also observed. Studies have shown that small crack widths are beneficial for the development of multiple cracks, which can effectively prevent water containing corrosive ions from entering the concrete interior, and thus, contributes to the improvement of durability and self-healing performance [45,46].

### 3.4. Fiber-Matrix Interface Performance

**Figure 10:**
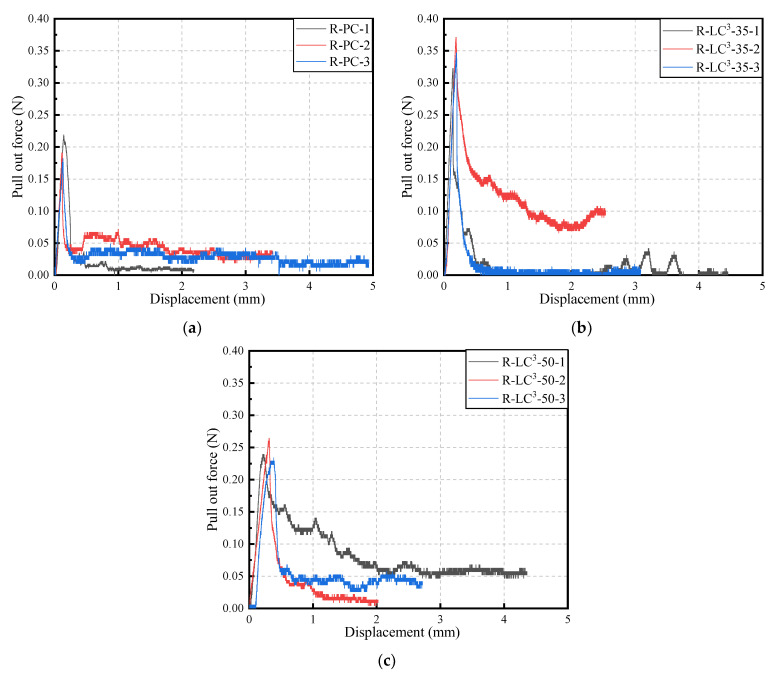
Single fiber pullout curves at 28 days: (**a**) R-PC; (**b**) R-LC^3^–35; (**c**) R-LC^3^–50.

Figure 10 shows the force-displacement curves of single fiber pull-out tests. Since PE fibers are hydrophobic fibers, the chemical bond between fibers and matrix can be ignored. Therefore, the bond strength between PE fibers and matrix is considered as pure frictional stress. The frictional stress *τ*_0_ can be calculated by the following formula [47]:

(1) $$\tau_{0}=\frac{P_{max}}{\pi d_{f}L_{f}\left(1+\eta \right)}\approx \frac{P_{max}}{\pi d_{f}L_{f}},$$

where *P_max_* is the peak pull-out force recorded, *d_f_* is the fiber diameter, *L_f_* is the fiber embedment length, and *η* is the ratio of effective fiber stiffness to matrix stiffness, which is assumed to be zero in this case [48].

The values of *τ*_0_ for different mix proportions are summarized in Table 7. The interfacial bond strengths of R-PC, R-LC^3^-35, and R-LC^3^-50 at 28 days are 0.419 MPa, 0.736 MPa, and 0.469 MPa, respectively. *τ*_0_ shows an initial increasing trend, followed by a decreasing trend. The tendency of *τ*_0_ corresponds with the development trends of the tensile strength of ECC. The frictional bond strength of the RFA groups is generally larger than that of the quartz sand group [43], which is due to the fact that the anhydrous phases in RFA could participate in the hydration process and that the angular shapes of RFA favor the interlocking effect between fiber and matrix. However, compared to R-LC^3^-35, the relatively lower *τ*_0_ of R-LC^3^-50 is more conducive to the pull-out of fibers and favorably leads to the development of fiber bridging capacity.

### 3.5. XRD Results

**Figure 11:**
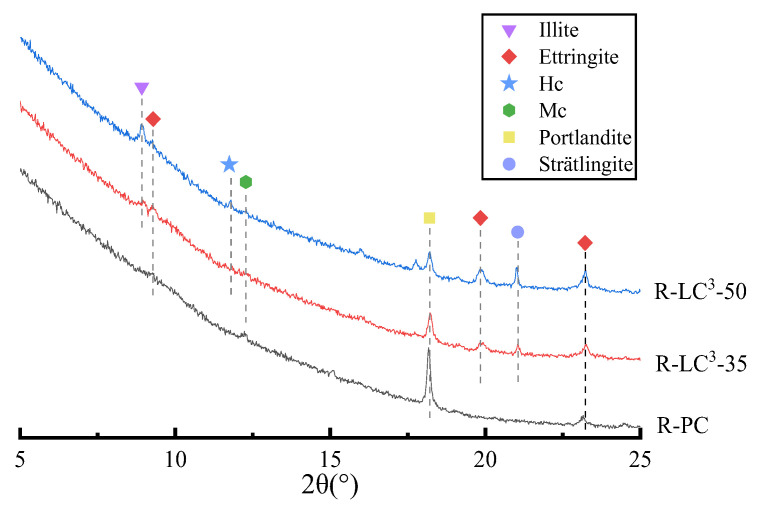
XRD patterns.

Figure 11 shows the XRD results of all samples at 28 days. After replacing cement with LC^3^, the intensity of the CH peak is significantly decreased, and the decrease in ratio increases with the LC^3^ substitution ratio. Mono-(Mc) and hemi-carboaluminates (Hc) peaks were also generated, owing to the reaction between calcium carbonate in limestone and the aluminates in both calcined clay and cement, as shown in Figure 10. The formation of additional phases could fill in the pores and the interstices between microparticles, and thus, densifies the microstructure of ECC matrix. The CaCO_3_ peak could be clearly distinguished in the LC^3^ matrix, which is due to the addition of extra limestone powder. The usage of limestone gives rise to the stabilization of ettringite, the peak of which is clearly observed in LC^3^ groups. Moreover, the AFm-type phase of stratlingite is also generated owing to the incorporation of LC^3^.

### 3.6. Environmental Impact Assessment

**Figure 12:**
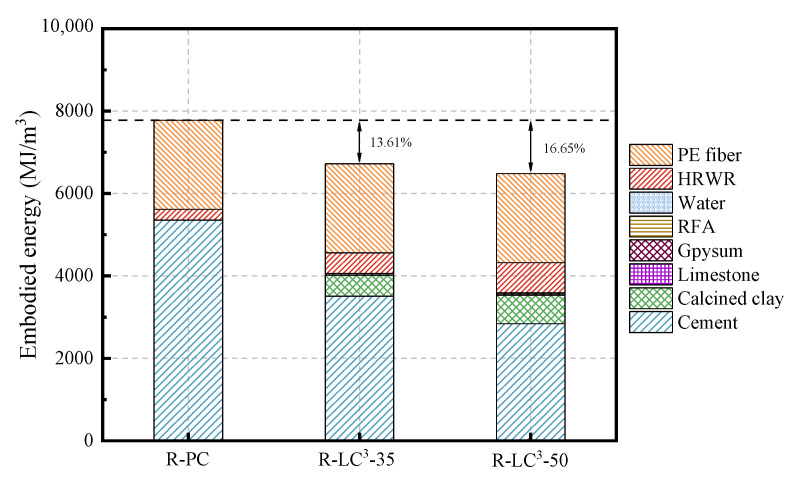
Summarization of the consumed energy by each component of ECC.

**Figure 13:**
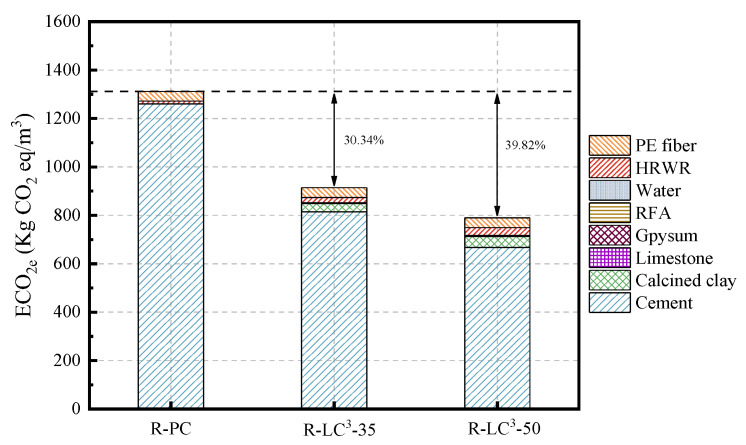
Summary of the GWP by each component of ECC.

**Figure 14:**
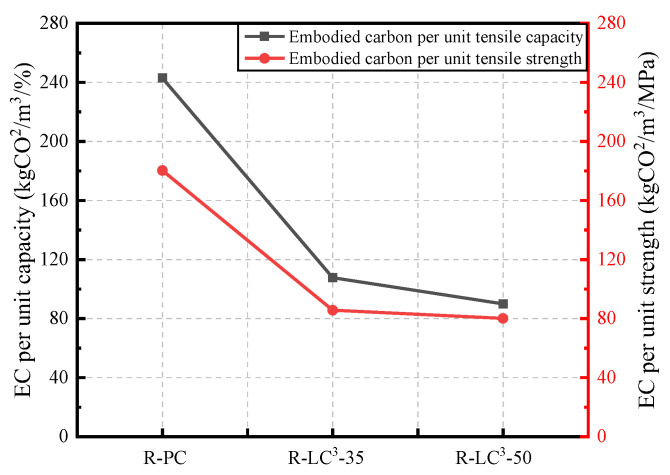
Comparison of the energy consumption of ECC per unit of tensile strain and per unit of tensile strength.

**Figure 15:**
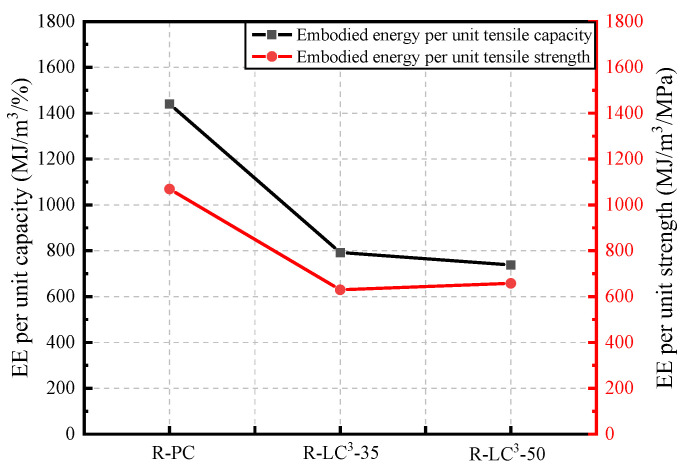
Comparison of the equivalent CO_2_ emission of ECC per unit of tensile strain and per unit of tensile strength.

The life cycle assessment (LCA) data for cement, calcined clay, gypsum, limestone, water, and HRWR used in this study were obtained from the GaBi software database. As the GaBi software lacked the data about efficient water reducers, recycled aggregates, and PE fibers, an equivalent model was established using data from references [25,41,42]. The Environmental Footprint 2.0 method in GaBi software was used to calculate two environmental impact indicators, namely energy consumption and global warming potential (GWP). The obtained results are shown in Figure 12, Figure 13, Figure 14 and Figure 15.

As seen from Figure 12 and Figure 13, the incorporation of LC^3^ significantly reduces the embodied energy (EE) and the equivalent CO_2_ (ECO_2e_). Of the two indicators, the reduction ratio of ECO_2e_ is more significant. Among all the mix proportions, cement is the material with the highest proportion of EE and ECO_2e_, indicating that cement is a highly polluting and energy-intensive material. In the control group, cement accounts for 68.79% of EE and 96.04% of ECO_2e_. The reduction ratios of EE for R-LC^3^-35 and R-LC^3^-50 are 13.61% and 16.65%, respectively, and the decreasing ratios of ECO_2e_ are 30.34% and 39.82%, respectively. In addition, the difference of carbon emission and energy consumption by RFAs and quartz sand is not significant. Since the recycled aggregate has high water absorption ratio, more water reducers are needed, which will slightly increase energy consumption and CO_2_ emissions. However, the impacts are very limited. Overall, cement is the main factor affecting energy consumption and CO_2_ emissions, and the addition of LC^3^ can remarkably reduce the impact of ECC on the environment.

Figure 14 and Figure 15 show the ECO_2e_ and EE per unit of tensile strength and tensile strain at 28 days. The addition of LC^3^ significantly reduces the EE and ECO_2e_ per unit of tensile strength and tensile strain. While, the reduction ratio is significant when the replacement ratio of LC^3^ is 35%, there is no obvious additional difference when the replacement ratio reaches 50%. From the perspective of unit tensile strength or unit tensile strain, the environmental impact of ECC after incorporating LC^3^ is remarkably reduced, and the comprehensive properties of ECC are improved.

### 3.7. Comprehensive Performance Evaluation

**Figure 16:**
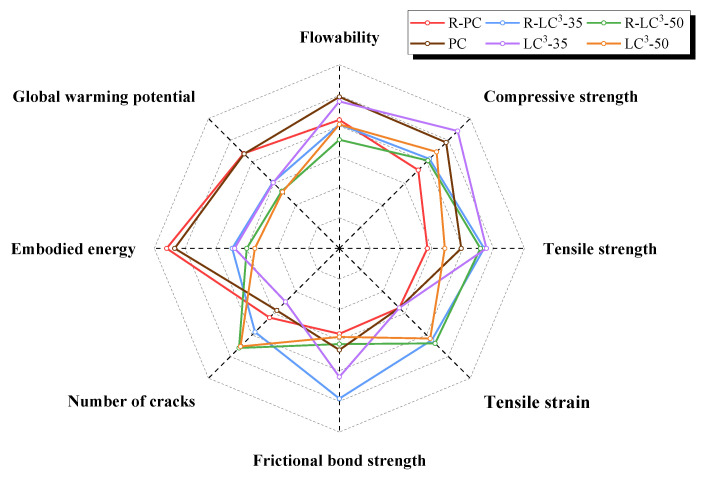
Comprehensive performance evaluation of RFA-LC^3^-ECC.

Based on previous research work from our group [43], a comprehensive evaluation of the performance of LC^3^-ECC incorporating RFA or quartz sand was conducted in terms of macroscopic mechanical performance indicators and environmental impact assessment, as shown in Figure 16. It can be observed that the addition of LC^3^ and RFA reduces the flowability of ECC. This is because the calcined clay in the LC^3^ system and the RFA have larger specific surface area and higher water absorption rate compared to cement and natural quartz sand. The addition of these two materials reduces the flowing of free water in the ECC system, resulting in decreased fluidity. The compressive strength of the ECC group with recycled aggregate at 28 days is lower than that of the quartz sand group due to the fact that the strength of RFA is inferior to that of quartz sand. However, it is worth noting that the partial replacement of OPC by LC^3^ helps to improve the compressive strength. Both ECC groups with recycled and natural aggregate achieved the highest strength when the LC^3^ replacement ratio was 35%. In addition, when the LC^3^ replacement ratio was 50%, the change trend of the compressive strength of ECC using RFA was opposite to that of ECC using natural aggregate. This could be explained by the fact that the unhydrated phases in RFA reacted during the new hydration process, resulting in increased strength.

With regard to the tensile performance at 28 days, both the ECC using RFA and ECC containing quartz sand achieved the maximum tensile strength at the LC^3^ replacement ratio of 35%. For the 50% LC^3^ replacement ratio, both types of ECC using recycled and natural aggregates exhibited the highest ductility. The addition of LC^3^ improves the bonding performance between fiber and matrix. Moreover, the interaction between RFA and LC^3^ increases the ductility of ECC, manifested by an increase in tensile strain and the number of cracks. The high toughness of RFA may enhance the interlocking effect between fiber and matrix, and the self-cementing properties of RFA also contributes to the densification of matrix. Regarding the energy consumption and carbon emissions, the natural aggregate ECC showed slightly lower values than that of ECC using recycled aggregate, which is due to the higher water absorption of RFA. A larger amount of water reducing agent is needed in order to achieve the same flowability. However, this difference is rather limited.

In summary, the replacement of OPC by LC^3^ is beneficial for improving the compressive strength and the tensile ductility of ECC. Meanwhile, LC^3^-ECC shows significant advantages over traditional ECC in terms of reducing environmental impacts. The addition of recycled aggregates may lower the compressive strength of ECC to some extent due to the intrinsic defects of recycled material, but the interaction between RFA and LC^3^ could further improve the ductility of ECC, with minimal impact on environment. Therefore, it can be concluded that the sustainable RFA-LC^3^-ECC has good environmental benefits while also achieving comparable mechanical properties.

## 4. Conclusions

In this study, the properties of RFA-LC^3^-ECC were comprehensively investigated through macro-mechanical testing, single fiber pull-out testing, XRD analysis, and environmental impact assessment. The sustainable ECC exhibits good mechanical properties as well as obvious environmental benefits. The following conclusions were drawn:

(1) Compared to RFA-ECC, the incorporation of LC^3^ increases the compressive strength, tensile strength, and tensile strain capacity. The improvement of strength could be explained by the formation of additional hydrates due to the pozzolanic activity of calcined clay as well as the refined pore structure of ECC matrix. The frictional bond strength between fiber and matrix also increases with addition of LC^3^.
(2) At the age of 150 days, the tensile stress-strain curves of ECC containing LC^3^ and RFA shifted from a bilinear model to a trilinear model. The hydrophobic PE fiber exhibited hydrophilic bonding performance when embedded in RFA-LC^3^-ECC matrix. This phenomenon is due to the fact that the self-cementing properties of RFA lead to the formation of additional hydration products and that the frictional bonding between fiber and matrix is enhanced. The tensile ductility of LC^3^-ECC incorporating RFA is higher than that of LC^3^-ECC using quartz sand.
(3) Environmental assessment revealed that the incorporation of LC^3^ significantly reduces the energy consumption and carbon emission. Although the usage of RFA slightly increases the values of EE and ECO_2e_ due to the requirement of more water reducer, this impact is rather limited. By comparing the EE and ECO_2e_ of unit tensile strength and unit tensile strain, the environmental impact of ECC incorporating LC^3^ and RFA is remarkably reduced, and the comprehensive properties of ECC are improved.

## Figures and Tables

**Table 1:** Mixture proportion of ECC (kg/m^3^).

Mixture Type	Cement	Calcined Clay	Gypsum	Effective Water	Limestone	RFA	PE	HRWRA
R-PC	1382	0	0	328	0	500	20	9
R-LC^3^-35	905	304	21	328	152	500	20	17
R-LC^3^-50	732	415	28	328	207	500	20	25

**Table 2:** Chemical composition of cementitious binders *.

Property	Cement	Calcined Clay	Gypsum	Limestone
MgO (%)	1.775	0.307	3.394	0.769
Na_2_O (%)	0.281	-	-	-
Al_2_O_3_ (%)	3.509	39.405	5.828	0.130
SiO_2_ (%)	15.406	53.732	14.046	0.309
P_2_O_5_ (%)	0.065	0.037	0.050	-
SO_3_ (%)	4.212	0.087	35.867	-
K_2_O (%)	0.0950	4.229	1.403	0.040
CaO (%)	69.862	0.102	37.048	98.715
Fe_2_O_3_ (%)	3.741	2.056	2.032	-
CuO (%)	0.029	-	-	-
ZnO (%)	0.100	-	-	-
SrO (%)	0.070	-	0.333	0.037
Rb_2_O (%)	-	0.037	-	-
Y_2_O_3_ (%)	-	0.001	-	-
ZrO_2_ (%)	-	0.008	-	-

* XRF analysis carried out by ZSX Primus II X-Ray Fluorimeter, Rigaku.

**Table 3:** Properties of PE fibers *.

Property	Value
Length *Lf*, mm	18
Diameter *df*, µm	25
Aspect ratio *Lf*/*df*	720
Fiber strength, MPa	2900
Modulus of elasticity, GPa	116
Specific gravity, g/cm^3^	0.97
Melting temperature, °C	150

* Provided by the supplier of PE fiber, the company QUANTUMETA in Beijing, China.

**Table 4:** LCA calculation data from the reference literature.

Material	Data for Each Material	
	EE (MJ/kg)	ECO_2e_ (kg CO_2_ eq/kg)
Superplasticizer [41]	29.42	1.309
RFA [25]	0.0034	0.0003
PE [42]	108	2.0

**Table 5:** Flowability of recycled aggregate ECC.

Mixture ID	Average Value (mm)
R-PC	144
R-LC^3^–35	141
R-LC^3^–50	134

**Table 6:** Cracking characteristics.

Sample	R-PC-28d	R-LC^3^-35-28d	R-LC^3^-50-28d	R-PC-150d	R-LC^3^-35-150d	R-LC^3^-50-150d
*N_c_*	48 ± 4	58 ± 3	69 ± 7	35 ± 4	44 ± 3	53 ± 6
*W_c_*/μm	93 ± 7	117 ± 6	103 ± 9	137 ± 15	110 ± 7	86 ± 9
*S_c_*/mm	1.68 ± 0.14	1.38 ± 0.07	1.17 ± 0.12	2.32 ± 0.27	1.83 ± 0.13	1.53 ± 0.17

**Table 7:** Frictional bond strength *τ*_0_. (Unit: MPa).

Mixtures ID	Average Value	Standard Deviation
R-PC	0.419	0.041
R-LC^3^–35	0.736	0.052
R-LC^3^–50	0.469	0.057

## Data Availability

The data presented in this study are available on request from the corresponding author.
